# Transcriptome sequencing of a chimaera reveals coordinated expression of anthocyanin biosynthetic genes mediating yellow formation in herbaceous peony (*Paeonia lactiflora* Pall.)

**DOI:** 10.1186/1471-2164-15-689

**Published:** 2014-08-19

**Authors:** Daqiu Zhao, Yao Jiang, Chuanlong Ning, Jiasong Meng, Shasha Lin, Wen Ding, Jun Tao

**Affiliations:** Key Laboratory of Crop Genetics and Physiology of Jiangsu Province, College of Horticulture and Plant Protection, Yangzhou University, Yangzhou, 225009 Jiangsu P.R. China

**Keywords:** Anthocyanins, Flower colour, Flavonoids, Herbaceous peony, Transcriptome, Yellow

## Abstract

**Background:**

Herbaceous peony (*Paeonia lactiflora* Pall.) is a traditional flower in China and a wedding attractive flower in worldwide. In its flower colour, yellow is the rarest which is ten times the price of the other colours. However, the breeding of new yellow *P. lactiflora* varieties using genetic engineering is severely limited due to the little-known biochemical and molecular mechanisms underlying its characteristic formation.

**Results:**

In this study, two cDNA libraries generated from *P. lactiflora* chimaera with red outer-petal and yellow inner-petal were sequenced using an Illumina HiSeq™ 2000 platform. 66,179,398 and 65,481,444 total raw reads from red outer-petal and yellow inner-petal cDNA libraries were generated, which were assembled into 61,431 and 70,359 Unigenes with an average length of 628 and 617 nt, respectively. Moreover, 61,408 non-redundant All-unigenes were obtained, with 37,511 All-unigenes (61.08%) annotated in public databases. In addition, 6,345 All-unigenes were differentially expressed between the red outer-petal and yellow inner-petal, with 3,899 up-regulated and 2,446 down-regulated All-unigenes, and the flavonoid metabolic pathway related to colour development was identified using the Kyoto encyclopedia of genes and genomes database (KEGG). Subsequently, the expression patterns of 10 candidate differentially expressed genes (DEGs) involved in the flavonoid metabolic pathway were examined, and flavonoids were qualitatively and quantitatively analysed. Numerous anthoxanthins (flavone and flavonol) and a few anthocyanins were detected in the yellow inner-petal, which were all lower than those in the red outer-petal due to the low expression levels of the phenylalanine ammonialyase gene (*PlPAL*), flavonol synthase gene (*PlFLS*), dihydroflavonol 4-reductase gene (*PlDFR*), anthocyanidin synthase gene (*PlANS*), anthocyanidin 3-*O*-glucosyltransferase gene (*Pl3GT*) and anthocyanidin 5-*O*-glucosyltransferase gene (*Pl5GT*).

**Conclusion:**

Transcriptome sequencing (RNA-Seq) analysis based on the high throughput sequencing technology was an efficient approach to identify critical genes in *P. lactiflora* and other non-model plants. The flavonoid metabolic pathway and glucide metabolic pathway were identified as relatived yellow formation in *P. lactiflora*, *PlPAL*, *PlFLS*, *PlDFR*, *PlANS*, *Pl3GT* and *Pl5GT* were selected as potential candidates involved in flavonoid metabolic pathway, which inducing inhibition of anthocyanin biosynthesis mediated yellow formation in *P. lactiflora*. This study could lay a theoretical foundation for breeding new yellow *P. lactiflora* varieties.

**Electronic supplementary material:**

The online version of this article (doi:10.1186/1471-2164-15-689) contains supplementary material, which is available to authorized users.

## Background

With the rapid development of the national economy and increasing integration in the international community, the demand for flowers is changing from high yield to high quality in recent years. Among the many qualities that influence flowers, flower colour is one of the most important indicators that determines its ornamental quality. Ornamental plants with novel flower colours can effectively stimulate the senses and psychology of consumers, as well as have a broader market appeal [1], such as the blue rose (*Rosa rugosa* Thunb.) and yellow violet (*Matthiola incana* R. Br.) [2]. Thus, breeding new flower cultivars with rare colours has been an important target for flower breeders.

Herbaceous peony (*Paeonia lactiflora* Pall.) is a traditional flower in China that belongs to the Paeoniaceae family and has been a well-known symbol of prosperity. In China, it enjoys a high status as a traditional flower and has shared the name with “The king and minister of flowers” with the tree peony (*Paeonia suffruticosa* Andr.) [3]. Until recently, there has been a large germplasm resource of *P. lactiflora* with more than 600 cultivars worldwide, and the flower colour characteristics vary widely among different cultivars, which can be divided into nine flower colour categories, i.e., red, pink, white, blue, purple, green, yellow, black and double colour [4]. Among these flower colours, yellow, which represents gold, suggests riches and honour, as well as reveals the king demeanour in flowers. In addition, the commercial price of yellow *P. lactiflora* cultivars is usually approximately 10 times more than purple or red cultivars. Whereas, yellow cultivars of *P. lactiflora* are very rare, where currently, there is only one pure yellow cultivar, namely ‘Huangjinlun’ [5]. This limitation can not highly meet the needs of consumers. Thus, breeding new *P. lactiflora* cultivars with yellow flowers has been an important aim, although it has been challenging using selective breeding and hybridisation breeding. Therefore, understanding of the biochemical and molecular mechanisms of yellow characteristic formation in *P. lactiflora*, identifying its key genes, and the ability to modify flower colour using modern genetic engineering are important to breed new *P. lactiflora* varieties in the future. However, very limited genomic resources are available for *P. lactiflora*, with only 195 nucleotide sequences deposited in GenBank until 24 January 2014, which has restricted the development of modern breeding technologies.

Transcriptome sequencing (RNA-Seq) is a highly efficient and conventional molecular biology research method based on next-generation sequencing technology [6]. In recent years, the continuous development and improvement of this technology, combined with a more mature commercial operation, have provided new approaches and designs in the study of functional genomics of different plants and have made transcriptomics a new starting point and breakthrough point in plant research [7–9]. Compared with the gene chip technique, which is based on the premise of a large number of genes or molecular database information, RNA-Seq can be studied in non-model organisms without corresponding genome sequences as a reference [10–12]. Moreover, transcriptome study enables researchers to understand plant gene functions and structures at a specific stage and is more convenient to reveal the molecular mechanisms of specific biological processes [13]. Recently, RNA-Seq has become widely applied in many plants such as cucumber (*Cucumis sativus* L.) [14, 15], Chinese cabbage (*Brassica rapa* L.) [16], blueberry (*Vaccinium corymbosum* L.) [17], peach (*Prunus persica* L. Batsch) [18], camelina (*Camelina sativa* L. Crantz) [19], etc. To the best of our knowledge, colour formation has been intensely investigated in fruits using RNA-Seq, and some information has been obtained regarding Unigenes, such as sweet orange (*Citrus sinensis* L. Osbeck) [20] and Chinese bayberry (*Myrica rubra* Sieb. and Zucc.) [21]. However, in flowers, RNA-Seq has been used in in-depth studies of colour formation only in wintersweet (*Chimonanthus praecox* L.) [22] and safflower (*Carthamus tinctorius* L.) [23], and the flavonoid biosynthetic pathway has been annotated in these plants which is widespread in regulating flower colour formation of plants. In model plants, this biosynthetic pathway can be divided into three stages [24]. The first stage is the conversion of phenylalanine to coumarate-CoA by phenylalanine ammonialyase gene (*PAL*), cinnamate 4-hydroxylase gene (*C4H*) and 4-coumarate CoA ligase gene (*4CL*). The second stage is the formation of dihydroflavonol by one molecule of coumarate-CoA and three molecules of malonyl-CoA catalyzed by chalcone synthase gene (*CHS*), chalcone isomerase gene (*CHI*), flavanone 3-hydroxylase gene (*F3H*), flavonoid 3′-hydroxylase gene (*F3′H*), flavonoid 3′,5′-hydroxylase gene (*F3′5′H*) and flavonol synthase gene (*FLS*). The third stage is the formation of various anthocyanidins by dihydroflavonols catalyzed by dihydroflavonol 4-reductase gene (*DFR*) and anthocyanidin synthase gene (*ANS*). The synthesized anthocyanidins are then modified through a series of glycosylation and methylation steps to form stable anthocyanins catalyzed by anthocyanidin 3-*O*-glucosyltransferase gene (*3GT*), anthocyanidin 5-*O*-glucosyltransferase gene (*5GT*) and methyl transferase gene (*MT*). However, there has been limited related information on *P. lactiflora*, and these studies provide very useful references for the study of yellow formation in *P. lactiflora*.

It is well known that plant materials with obvious differences are essential in the study of gene function. Furthermore, genetic mutants that control relative characteristics are the best materials to study gene function. In the present study, a flower colour chimaera cultivar ‘Jinhui’ with a consistent genetic background red outer-petal and yellow inner-petal was used as the experimental material including four developmental stages (S1, flower-bud stage; S2, initiating bloom; S3, bloom stage; and S4, wither stage), and Illumina sequencing was adopted to analyse their difference of transcriptome. Based on this, the expression patterns of differentially expressed genes (DEGs) identified in the flavonoid biosynthetic pathway were analysed using real-time quantitative polymerase chain reaction (Q-PCR), and qualitative and quantitative analysis of flavonoids was performed using high-performance liquid chromatograph (HPLC)-electrospray ionization (ESI)-mass spectrometry (MS^n^) technology, thereby revealing the biochemical and molecular mechanisms of yellow formation in *P. lactiflora*. Taken together, these results would provide a foundation for breeding yellow *P. lactiflora* varieties.

## Results

### Colour indices

**Figure 1:**
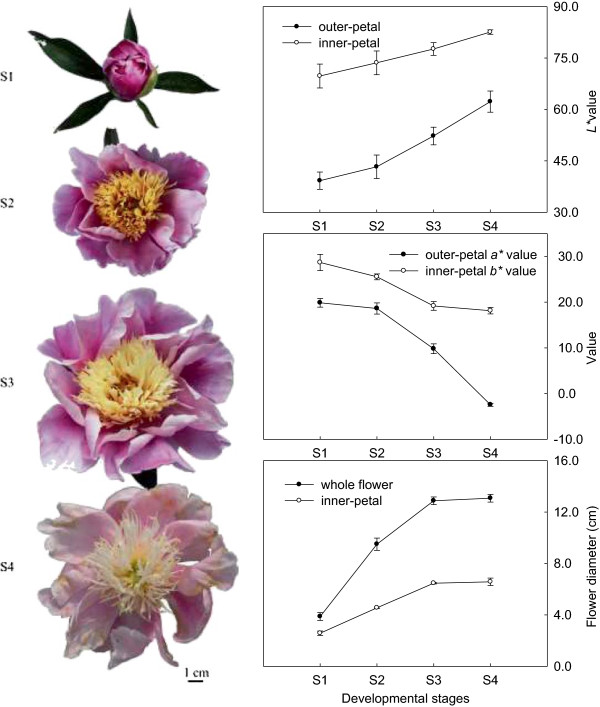
**Figures of** ***P. lactiflora*** **flower developmental stages, flower qualitative trait and color variables.** S1 (Stage 1), flower-bud stage; S2 (Stage 2), initiating bloom stage; S3 (Stage 3), bloom stage; S4 (Stage 4), wither stage.

To obtain the same genetic background, a flower colour chimaera cultivar ‘Jinhui’ with red outer-petal and yellow inner-petal was used to study the mechanism of yellow formation in *P. lactiflora* (Figure 1). Firstly, their colour indices were measured. On one hand, the flower colours were defined according to the Royal Horticultural Society Color Chart (RHSCC). During flower development, the colour gradation of the outer-petal ranged from 164A, 73A and 75B to 75C, while that of the inner-petal changed from 11A, 11B and 11C to 11D. On the other hand, flower colours were expressed as L^*^, a^*^ and b^*^. In a uniform colour space, L^*^ represented lightness, a^*^ represented the ratio of red/magenta and green, and b^*^ represented the ratio of yellow and blue [25]. In four different developmental stages, L^*^ values of the outer-petal and inner-petal were all gradually increased which was always lower in the outer-petal compared to the inner-petal; however, the b^*^ value of the outer-petal and a^*^ value of the inner-petal decreased, which all tended to zero. These colour indices showed that the colours of the outer-petal and inner-petal were pure red and yellow in S1, respectively, and then faded gradually with flower development. In addition, the flower diameter was also measured, such that the whole flower and inner-petal were similar and had constantly increased from S1 to S4. Taken together, these measured results were consistent with the visual results.

### RNA-Seq and de novo assembly

To deeply elucidate the molecular mechanism underlying yellow colour formation, RNA-Seq analysis were performed using the Illumina platform. Each library of the outer-petal and inner-petal produced 66,179,398 and 65,481,444 total raw reads, following cleaning and quality checks, and 60,234,256 and 61,296,620 total clean reads were obtained with a single read length of 90 nt and a Q20 percentage (proportion of nucleotides with quality value larger than 20 in reads) over 98.50% (Table 1). These data showed that the RNA-Seq quality was better, which can be used for further analysis.

**Table 1:** **Output statistics of RNA-Seq of** ***P. lactiflora*** **outer-petal and inner-petal**

Samples	Total raw reads	Total clean reads reads	Total clean nucleotides	Q20 percentage	N percentage	GC percentage
outer-petal	66,179,398	60,234,256	5,421,083,040	98.52%	0.00%	45.76%
inner-petal	65,481,444	61,296,620	5,516,695,800	98.58%	0.00%	45.22%

Subsequently, short reads from the outer-petal and inner-petal were assembled into 107,199 and 127,518 Contigs, which contained an average length of 360 and 337 nt, together with N50 length (covering 50% all the nucleotide sequences of the largest Contig length) of 745 and 664 nt, respectively. After linking the Contigs together, 61,431 and 70,359 Unigenes with an average length of 628 and 617 nt of the outer-petal and inner-petal were identified, respectively, and their N50 values were all 1047 nt. Finally, 61,408 non-redundant All-unigenes were obtained according to splicing and reducing the redundancy of the Unigenes sequence in the outer-petal and inner-petal, and its average length and N50 value were 763 nt and 1219 nt, respectively (Table 2). Among these, there were 32,517 Unigenes with a length of 100 to 500 nt, which accounted for 52.92%, and excluding N rate reached 100%. All these results showed that the assembly quality was sufficiently high for gene function analysis. In addition, the distributions of the reads in All-unigenes were analysed, and these results showed that the number of reads in the 3′ and 5′ ends from All-unigenes was relatively low, particularly in the 3′ and 5′ tail ends. However, the number of reads located in 0.2 to 0.8 was relatively high, and its distribution was balanced (Additional file 1: Figure S1).

**Table 2:** **Data assembly of transcriptome Contig and Unigene in** ***P. lactiflora*** **outer-petal and inner-petal**

	Sample	Total number	Total length (nt)	Mean length (nt)	N50	Total consensus sequences	Distinct clusters	Distinct singletons
Contig	outer-petal	107,199	38,543,748	360	745	-	-	-
	inner-petal	127,518	42,986,411	337	664	-	-	-
Unigene	outer-petal	61,431	38,554,638	628	1047	61,431	16,143	45,288
	inner-petal	70,359	43,390,011	617	1047	70,359	20,147	50,212
	All	61,408	46,873,884	763	1219	61,408	20,191	41,217

### Functional annotation

Due to the lack of a complete genome sequence in *P. lactiflora*, 61,408 All-unigenes were blasted against six public databases, i.e., non-redundant protein database (NR), Swiss-Prot protein database (Swiss-Prot), Kyoto encyclopedia of genes and genomes database (KEGG), cluster of orthologous groups of proteins database (COG), gene ontology database (GO) and non-redundant nucleotide database (NT). These results revealed that 37,511 All-unigenes were annotated, which accounted for 61.08%. Of the All-unigenes, 35,972, 30,199 and 26,674 All-unigenes could be annotated to NR, NT and GO databases, accounting for 58.57%, 49.17% and 43.43%, respectively, whereas in the Swiss-Prot, KEGG and COG databases, only 22,655, 20,294 and 13,089 All-unigenes were annotated, which accounted for 36.89%, 33.04% and 21.31%. For the NR annotation, the ratio of the All-unigenes distributed in 10^-15^ < e-value < 10^-5^ and 0 < e-value < 10^-100^ with 17.4% and 16.9% was maximum; 67.4% of All-unigenes showed 60-95% similarity; and 30,627 All-unigene sequences shared a similarity with available plant sequences from *Vitis vinifera*, *P. persica*, *Ricinus communis*, *Populus trichocarpa*, *Fragaria vesca* subsp. Vesca, *C. sativus* and *Glycine max*, including up to 48.5% similarity with *Vitis vinifera* (Additional file 2: Figure S2).

**Figure 2:**
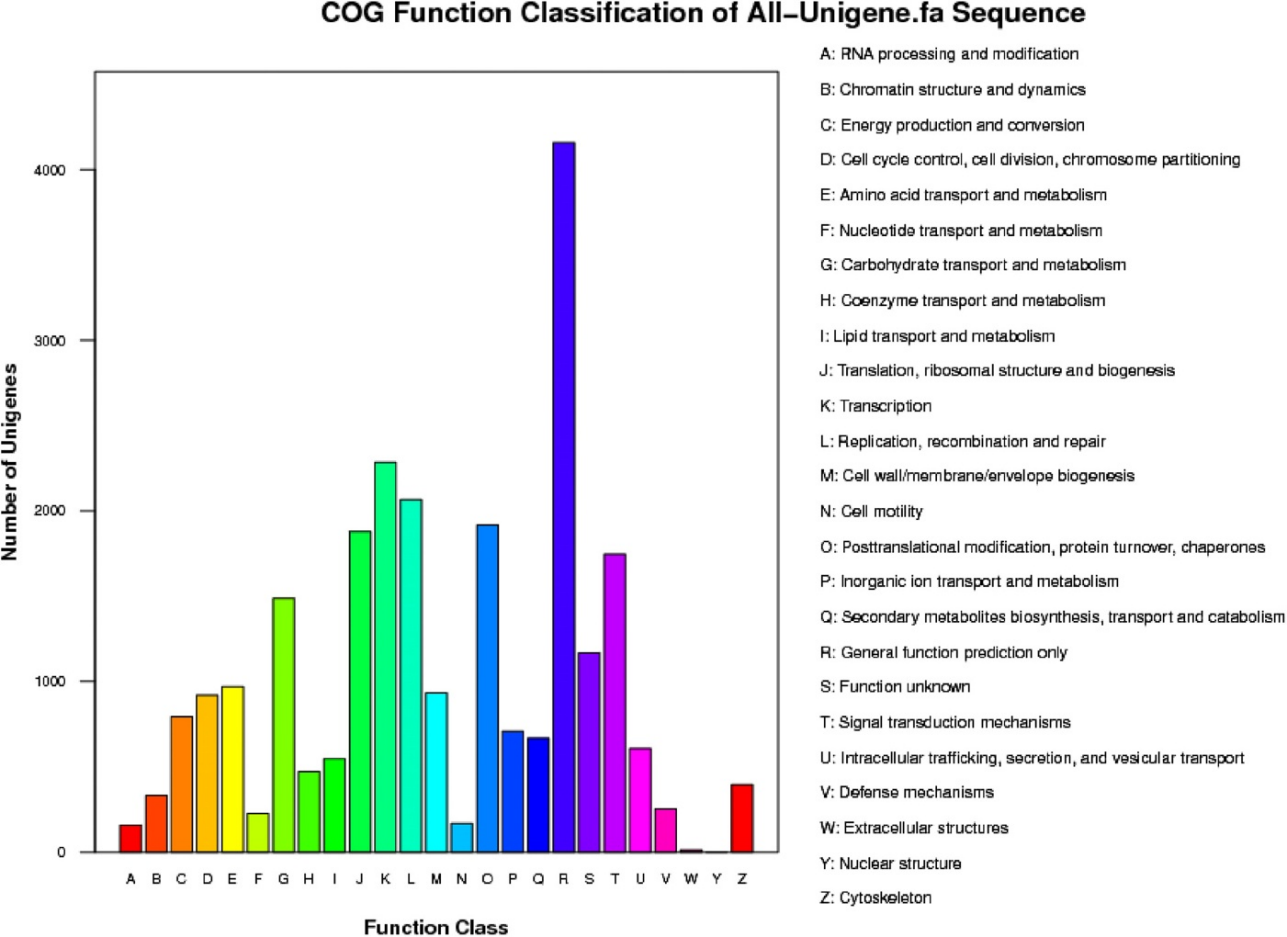
**Figure of COG categories of the annotated All-unigenes.**

When compared with the COG database, 13,089 All-unigenes were divided into 25 different functional classifications, and the functional types comprehensively involved most of the life activities (Figure 2). Among these types, the maximum number of All-unigenes was categorised as general function prediction only (4,158 All-unigenes, 16.71%), followed by transcription (2,284 All-unigenes, 9.18%), replication, recombination and repair (2,067 All-unigenes, 8.31%) and posttranslational modification, protein turnover, chaperones (1,917 All-unigenes, 7.70%), while the minimum number was classified to nuclear structure (2 All-unigenes, 0.008%).

### Comparative analysis of DEGs and GO functional annotation between the outer-petal and inner-petal

**Figure 3:**
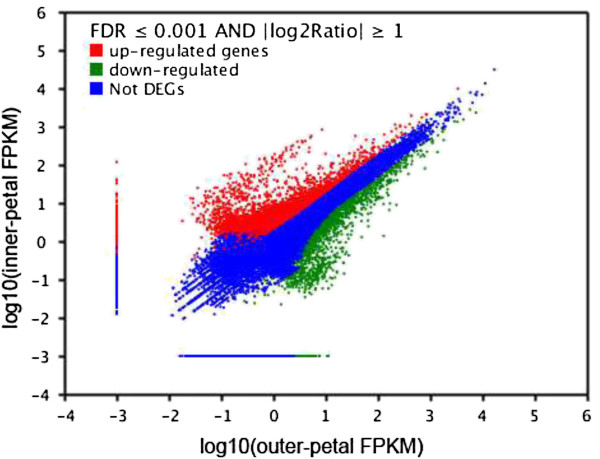
**Figure of DEGs in** ***P. lactiflora*** **outer-petal and inner-petal.** Red scatters indicate up-regulated DEGs, green scatters indicate down-regulated DEGs, and blue scatters indicate no difference DEGs in expression between the outer-petal and inner-petal.

**Figure 4:**
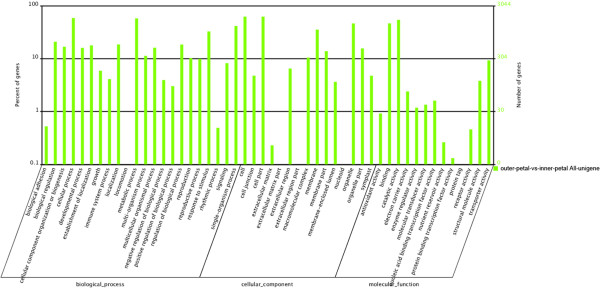
**Figure of GO categories of DEGs in** ***P. lactiflora*** **outer-petal and inner-petal.**

To identify key genes controlling yellow formation in *P. lactiflora*, the transcriptomes of the outer-petal and inner-petal were comparatively analysed based on the outer-petal as the control group. As shown in Figure 3, 6,345 DEGs were obtained, among which, the expression levels of 3,899 DEGs (red scatters) were up-regulated and the expression levels of 2,446 DEGs (green scatters) were down-regulated. Furthermore, abundant blue scatters indicated no difference in expression between the outer-petal and inner-petal. On the basis of sequence homology, these 6,345 DEGs could be classified into three categories in GO assignments: biological process, cellular component and molecular function, and they embraced 22, 15 and 13 functional classifications, respectively (Figure 4). In the biological process category, cellular process (1,786 DEGs, 15.74%) and metabolic process (1,758 DEGs, 15.49%) accounted for the major proportion. In the cellular component category, 1,894 and 1,894 DEGs (23.37%) were enriched in the cell and cell part. Under the molecular function category, catalytic activity (1,649 DEGs, 44.41%) and binding (1,412 DEGs, 38.03%) were dominant functions.

### Functional classification using KEGG

**Figure 5:**
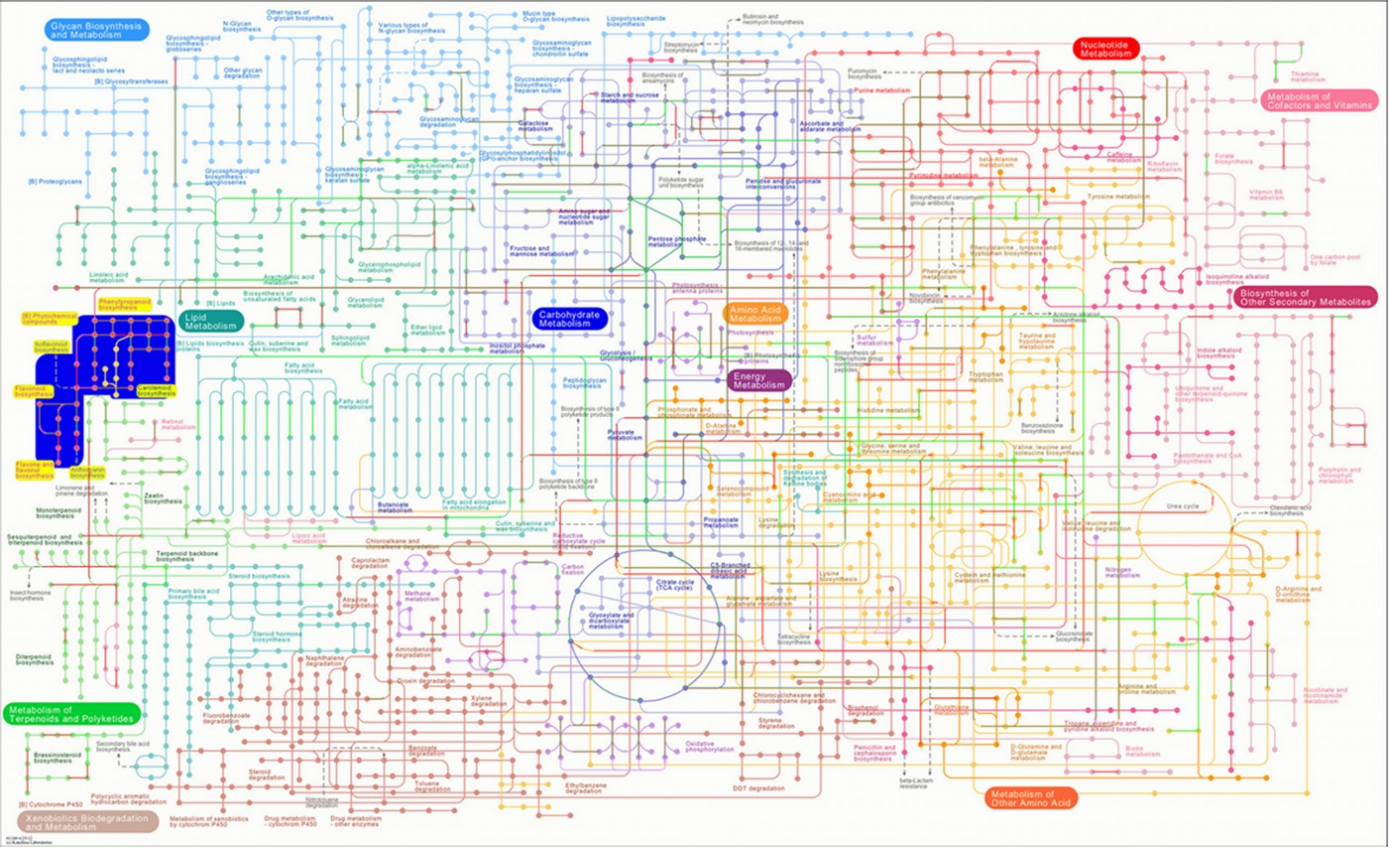
**Interactive pathways during the outer-petal and inner-petal development.** Blue part is flavonoid metabolic pathway related to colour development.

The pathway database was the major component of KEGG, which contained most of the metabolic pathways representing information on the molecular interaction and reaction networks [26]. In this study, 6,345 DEGs were subsequently analysed in the KEGG pathway database, where 2,559 DEGs (40.33%) were assigned to 121 KEGG pathways and only 37 pathways met the condition of Q-value ≤ 0.05 (Figure 5, Additional file 3: Table S1). Among these pathways, metabolic pathways demonstrated the largest number of DEGs (796 DEGs, 31.11%, ko01100), followed by biosynthesis of secondary metabolites (429 DEGs, 16.76%, ko01110), plant-pathogen interaction (189 DEGs, 7.39%, ko04626), plant hormone signal transduction (156 DEGs, 6.1%, ko04075), endocytosis (146 DEGs, 5.71%, ko04144), starch and sucrose metabolism (145 DEGs, 5.67%, ko00500) and glycerophospholipid metabolism (142 DEGs, 5.55%, ko00564). Many studies revealed that colour development of the *P. lactiflora* petal was closely related to flavonoids [27–29], and our previous study also confirmed this conclusion [30]. As expected, five pathways related to colour development were identified, namely phenylpropanoid biosynthesis (82 DEGs, 3.2%, ko00940), flavonoid biosynthesis (43 DEGs, 1.68%, ko00941), flavone and flavonol biosynthesis (39 DEGs, 1.52%, ko00944), isoflavonoid biosynthesis (16 DEGs, 0.63%, ko00943) and anthocyanin biosynthesis (8 DEGs, 0.31%, ko00942), and they all belonged to the flavonoid metabolic pathway which could be establish connection taking flavonoid biosynthesis as the ligament.

### Expression analysis of candidate DEGs related to colour development

**Figure 6:**
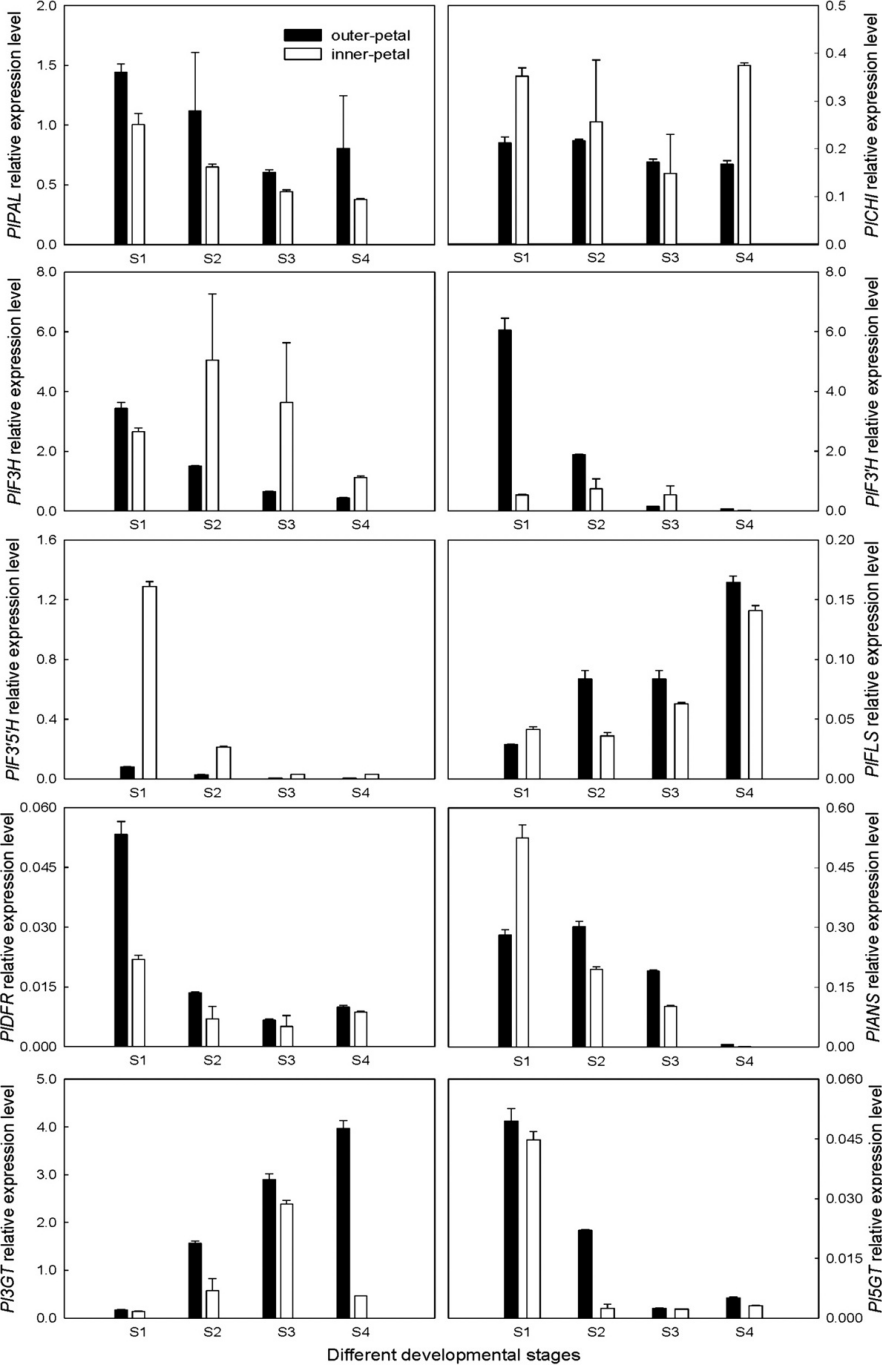
**Expression analysis of 10 candidate DEGs related to colour development in** ***P. lactiflora*** **.**

According to the genes annotated in KEGG and the results of flower colour formation obtained in many plants [31, 32], 10 DEGs were selected for expression analysis (Table 3). Among these DEGs, *PAL*, *CHI*, *F3H*, *F3′H*, *DFR*, *ANS*, *3GT* and *5GT* had been registered in GenBank, and their accession numbers were JQ070801, JN119872, JQ070802, JQ070803, JQ070804, JQ070805, JQ070806 and JQ070807, respectively. However, *F3′5′H* and *FLS* were first found in *P. lactiflora*. Among them, more than one Unigene was annotated as the same enzyme, which might be because these Unigenes represented different members of the same gene family, different fragments of a single transcript, or both, except *PAL* and *F3′5′H* [33]. As shown in Figure 6, Q-PCR was used to study their expression patterns in four different developmental stages of the outer-petal and inner-petal. Single gene expression level analyses indicated that although the expressions of all 10 DEGs were detected during flower development, but their expression levels were different. Among the 10 DEGs, the expression levels of *PlF3H* and *PlF3′H* were the highest, and downstream *PlDFR* and *Pl5GT* exhibited the lowest expression levels, while the other DEGs ranged between the two genes listed above. In terms of the overall trend, the expression levels of the 10 DEGs were similar during the outer-petal and inner-petal development except in some stages. For example, the expression levels of *PlFLS* and *Pl3GT* increased until they reached the maximum level during flower development, the other 8 DEGs all showed a downward trend, and they only had some differences in quantity between the outer-petal and inner-petal. For different petals, the first key gene in flavonoid metabolism *PlPAL*; *PlDFR* controlling the formation of leucoanthocyanidin; and *Pl3GT* together with *Pl5GT*, which catalyses the formation of the complex aglycone of anthocyanin composition, all had higher expression levels in the outer-petal compared to the inner-petal in the four different developmental stages, whereas *F3′5′H* showed an opposite trend.

**Table 3:** **Candidate DEGs involved in** ***P. lactiflora*** **colour development**

Gene	Enzyme	Enzyme no.	KO id	No. all	No. up	No. down
*PAL*	Phenylalanine ammonia-lyase	[EC: 4.3.1.24]	K10775	1	0	1
*CHI*	Chalcone isomerase	[EC: 5.5.1.6]	K01859	2	2	0
*F3H*	Flavanone 3-hydroxylase	[EC: 1.14.11.9]	K00475	2	1	1
*F3′H*	Flavonoid 3′-hydroxylase	[EC: 1.14.13.21]	K05280	14	5	9
*F3′5′H*	Flavonoid 3′,5′-hydroxylase	[EC: 1.14.13.88]	K13083	1	1	0
*FLS*	Flavonol synthase	[EC: 1.14.11.23]	K05278	6	1	5
*DFR*	Dihydroflavonol 4-reductase	[EC: 1.1.1.219]	K13082	3	2	1
*ANS*	Anthocyanidin synthase	[EC: 1.14.11.19]	K05277	4	0	4
*3GT*	Anthocyanidin 3-*O*-glucosyltransferase	[EC: 2.4.1.115]	K12930	3	3	0
*5GT*	Anthocyanidin 5-*O*-glucosyltransferase	[EC: 2.4.1.298]	K12338	5	1	4

No. All indicates the total number of DEGs, No. Up indicates the number of DEGs in the inner-petal compared with the outer-petal in S2, No. Down indicates the number of DEGs in the inner-petal compared with the outer-petal in S2.

### Qualitative and quantitative analysis of flavonoids

**Figure 7:**
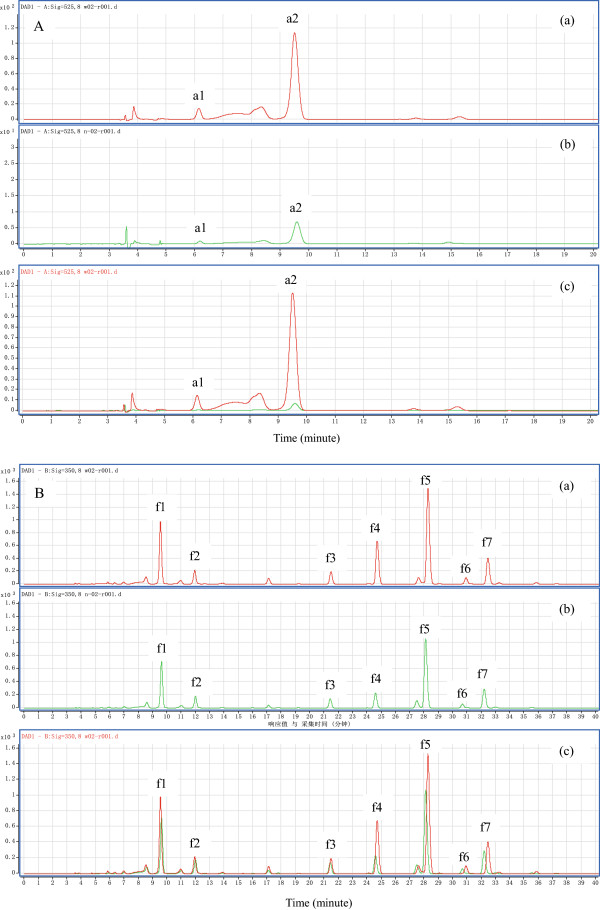
**HPLC chromatograms of** ***P. lactiflora*** **anthocyanins (A, detected at 525 nm) and anthoxanthins (B, detected at 350 nm) in S2. (a)** indicates HPLC chromatograms of outer-petal; **(b)** indicates HPLC chromatograms of inner-petal; **(c)** indicates HPLC chromatograms of **(a)** and **(b)** merge together. a1-a2 indicates identified anthocyanins; f1-f7 indicates identified anthoxanthins.

To examine whether flavonoid accumulation in *P. lactiflora* petals was consistent with the expression patterns of candidate DEGs related to colour development, qualitative and quantitative analysis of flavonoids were performed using HPLC-ESI-MS^n^ in the same samples used in the expression analysis. On the basis of the ultraviolet–visible absorption characteristics, anthocyanins, anthoxanthins and chalcones were detected under the wavelength of 525 nm, 350 nm and 365 nm, respectively. As shown in Figure 7, the chromatographic peaks of the outer-petal and inner-petal were identical, although there were only some differences in the peak area. The HPLC retention time and ultraviolet–visible spectral properties together with mass spectrometric data of identified main chromatographic peaks were listed in Additional file 4: Table S2. Moreover, two (a1 and a2) and seven (f1, f2, f3, f4, f5, f6 and f7) peaks were identified at 525 nm and 350 nm, namely cyanidin-3,5-di-*O*-glucoside, peonidin-3,5-di-*O*-glucoside, kaempferol di-hexoside, kaempferol-3-*O*-malonylglucoside-7-*O*-glucoside, quercetin-3-*O*-galactoside, luteolin-7-*O*-galloylglucoside, luteolin-7-*O*-glucoside, isorhamnetin-3-*O*-glucoside and flavone C-glycoside, respectively.

**Figure 8:**
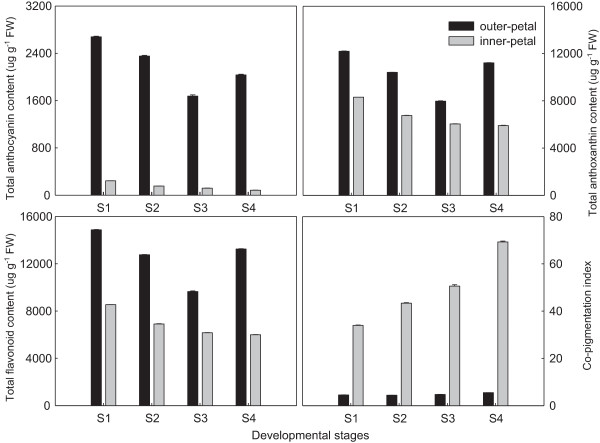
**Total content of anthocyanins, anthoxanthins and flavonoids, and co-pigmentation index in** ***P. lactiflora*** **outer-petal and inner-petal during flower development.**

Relevant standards were used as the references for the relative quantitative analysis of flavonoids, the results showed that there were tremendous differences in both the two different petals and also during different developmental stages of the same petals (Table 4). For the composition, peonidin-3,5-di-*O*-glucoside was the main component of anthocyanins in *P. lactiflora* outer-petal and inner-petal, which accounted for approximately 73.20% and 65.94% of the total anthocyanin contents in the outer-petal and inner-petal, respectively. In addition, the main component of anthoxanthins in *P. lactiflora* outer-petal and inner-petal was luteolin-7-*O*-glucoside, which comprised approximately 33.69% and 36.04% of the total anthoxanthin contents in the outer-petal and inner-petal, respectively, which was followed by kaempferol di-hexoside (17.45% and 19.41%), luteolin-7-*O*-galloylglucoside (16.14% and 9.21%) and flavone C-glycoside (9.99% and 10.93%). Concerning the content of composition, the results showed that the content of each composition in the outer-petal was basically higher compared to the inner-petal, particularly for a1 and a2 of anthocyanins. In S1, the contents of a1 and a2 in the outer-petal were 11 times as much as that of the inner-petal and increased to 30 times in S4. In the case of different developmental stages, each anthocyanin and anthoxanthin composition in the outer-petal were basically decreased from S1 to S3, and slightly increased in S4. However, the compositions in the inner-petal were always decreased with flower development, except f1, f4 and f7 in S4. For total content, the total contents of anthocyanins, anthoxanthins and flavonoids in the outer-petal were all higher compared to the inner-petal, and specifically, the difference in anthocyanins was achieved by a multiple of 10. Meanwhile, the total contents of anthocyanins, anthoxanthins and flavonoids in the outer-petal and inner-petal all presented a downward trend as flower development, although there was an increase in the outer-petal at S4. Furthermore, the co-pigment index (CI), which determined whether had the co-pigment effect between anthoxanthins and anthocyanins demonstrated an increasing tendency in both the outer-petal and inner-petal. In the outer-petal, the CI had increased from 4.55 in S1 to 5.51 in S4, in contrast, and its developmental difference in the inner-petal was more significant and increased from 34.00 in S1 to 69.28 in S4 (Figure 8).

**Table 4:** **Contents of anthocyanin and anthoxanthin compositions in** ***P. lactiflora*** **petals during flower development (ug g** ^**-1**^ **FW)**

Peak	S1		S2		S3		S4	
	outer-patal	inner-petal	outer-patal	inner-petal	outer-patal	inner-petal	outer-patal	inner-petal
a1	149.78 ± 0.96	12.46 ± 0.21	134.69 ± 0.90	7.40 ± 0.16	98.90 ± 1.92	5.72 ± 0.18	135.37 ± 1.28	3.64 ± 0.06
a2	1979.83 ± 0.34	171.44 ± 0.06	1719.72 ± 10.97	103.33 ± 0.25	1210.93 ± 26.70	84.84 ± 1.60	1490.26 ± 12.52	49.10 ± 0.71
f1	2202.62 ± 0.37	1517.01 ± 2.59	1812.72 ± 9.11	1317.25 ± 3.11	1280.69 ± 21.37	1198.32 ± 12.98	1991.88 ± 14.29	1208.84 ± 12.56
f2	560.75 ± 1.57	470.36 ± 0.73	459.87 ± 0.51	371.93 ± 1.14	359.03 ± 4.97	318.48 ± 2.51	497.65 ± 2.17	314.18 ± 2.74
f3	448.96 ± 0.29	421.65 ± 7.23	436.23 ± 0.98	311.22 ± 9.74	365.77 ± 3.66	263.00 ± 1.73	483.65 ± 1.93	191.72 ± 0.30
f4	1834.72 ± 14.07	558.33 ± 1.48	1599.16 ± 9.53	532.38 ± 2.31	1339.25 ± 8.09	599.89 ± 3.60	1968.99 ± 16.64	795.47 ± 3.37
f5	4303.42 ± 15.01	3088.60 ± 1.24	3609.96 ± 9.52	2494.02 ± 7.83	2607.99 ± 1.78	2181.54 ± 1.34	3554.77 ± 6.46	1968.36 ± 1.42
f6	244.87 ± 4.51	186.11 ± 2.51	225.65 ± 2.33	136.93 ± 0.73	187.03 ± 0.85	112.52 ± 1.28	255.83 ± 1.59	87.42 ± 1.37
f7	1175.88 ± 2.61	948.82 ± 9.70	1016.03 ± 3.68	718.43 ± 2.48	870.68 ± 16.73	627.85 ± 2.05	1110.19 ± 8.76	657.09 ± 19.84

S1 (Stage 1), flower-bud stage; S2 (Stage 2), initiating bloom stage; S3 (Stage 3), bloom stage; S4 (Stage 4), wither stage.

## Discussion

*P. lactiflora* is an important ornamental plant with a cultivation history of more than 4000 years in China. Due to abundant flower colours, variable flower shapes, sharp flower fragrances and attractive flower poses, *P. lactiflora* exhibits a very high ornamental value and rich landscaping application value, which has become increasingly favoured by people worldwide. However, compared with plants in which genome sequencing has been completed, such as arabidopsis (*Arabidopsis thaliana* (L.) Heynh.) [34], mei (*Prunus mume* Sieb. et Zucc.) [35], lotus (*Nelumbo nucifera* Gaertn.) [36], spider flower (*Tarenaya hassleriana* (Chodat) Iltis) [37] and carnation (*Dianthus caryophyllus* L.) [38], a weak molecular basis and an incompletely clear genetic background in *P. lactiflora* severely limit its in-depth study. Transcriptome refers to the identity of each expressed gene and its expression level for cells in a particular state, whose expression largely controls the phenotype of an organism. Unlike the genome, the transcriptome is more dynamic and provides more valuable reference information for cell function interpretation, but its study is more inexpensive [39]. Due to overcoming technological limitations, transcriptome studies have been extensively developed in plants [32, 40–42]. Moreover, according to a different purpose of the study, RNA-Seq can be divided into two methods: on one hand, different experimental materials are mixed equally to sequence, which can obtain the largest number of unique transcripts [21, 23]; on the other hand, different experimental materials are sequenced separately, which can isolate tissue-specific expression genes [21, 43, 44]. Wang et al. aimed to elucidate the transcriptome profiling of radish (*Raphanus sativus* L.) root and identify the critical gene response to lead (Pb) stress, two radish root cDNA samples, where one untreated control and the other Pb-stressed with Pb (NO_3_)_2_ at 1000 mg were separately sequenced, and 4,614 DEGs were obtained [45]. In this study, to characterize the transcriptome of *P. lactiflora* red outer-petal and yellow inner-petal and identify the critical genes mediating yellow formation, Illumina sequencing was firstly employed to the outer-petal and inner-petal with significantly different colours separately. Each library of the outer-petal and inner-petal produced 5,421,083,040 and 5,516,695,800 total clean nucleotides with a Q20 percentage over 98.50% in which the quantity and quality were all relatively high compared with other plants, such as the apple tree [46] and *M. rubra* [21]. Subsequently, 61,431 and 70,359 unigenes with an average length of 628 and 617 nt were found in the outer-petal and inner-petal, and 6,345 DEGs were identified, which provided a large amount of information for molecular biological research in *P. lactiflora*.

Colour formation is a complex process that includes many metabolic pathways and gene expression. In the transcriptome study of colour, Xu et al. speculated that enhanced photosynthesis and reduced downstream gene expression in the carotenoid biosynthetic pathway were the key reason for sweet orange red-flesh mutant lycopene accumulation, which controlled red formation [47]. Recently, two varieties of flesh in four developmental stages were used to perform RNA-Seq by this research group, which further confirmed the correlation between carotenoid metabolism and red colour formation in sweet orange red-flesh mutant [20]. In *M. rubra*, Feng et al. aimed to obtain a general overview of the transcriptome, four libraries containing mixed tissues, and three different developmental fruits were designed for RNA-Seq [21]. This study identified 41,239 Unigenes, generated interactive pathways during bayberry fruit ripening, and found that the expression levels of flavonoid biosynthetic genes were overall increased, which was consistent with flavonoid accumulation and more intense colour. To the best of our knowledge in flower colour, transcriptome studies had been performed only on *C. praecox* [42] and *C. tinctorius* [23]. In the transcriptome database of *C. praecox*, 31,142 Unigenes could be annotated in 266 different metabolic pathways. Furthermore, 105 Unigenes with an average length of 677 bp were associated with the metabolic pathway of flower colour, including the flavonoid biosynthetic pathway (70 Unigenes), flavone and flavonol biosynthetic pathway (24 Unigenes) and anthocyanin biosynthetic pathway (11 Unigenes), which was convenient for gene cloning and function identification [42]. Otherwise, the flavonoid biosynthetic pathway (274 Unigenes), flavone and flavonol biosynthetic pathway (114 Unigenes) and anthocyanin biosynthetic pathway (20 Unigenes) were identified according to the RNA-Seq comparison of red and white *C. tinctorius*. Moreover, the expression levels of 12 flavonoid biosynthetic pathway-related candidate Unigenes were analysed, indicating that *CHS* (Unigene96945) was very important for *C. tinctorius* flavonoid synthesis [23]. In the present study, 2,559 DEGs in the outer-petal and inner-petal had been annotated to 121 metabolic pathways, and there were 37 metabolic pathways enriched DEGs, which revealed the formation process complexity of different petals. Among these pathways, the flavonoid metabolic pathway containing phenylpropanoid biosynthesis, flavonoid biosynthesis, anthocyanin biosynthesis, isoflavonoid biosynthesis, flavone and flavonol biosynthesis was annotated, which validated the conclusion that flavonoids were the pigment of *P. lactiflora* petal [27, 28, 30]. Moreover, carotenoid biosynthesis and zeatin biosynthesis were also found in metabolic pathways, which might be resulted from incomplete petalody stamens. Interestingly, 7 glucide metabolic pathways, including pentose and glucuronate interconversions, starch and sucrose metabolism, glycolysis/gluconeogenesis, other glycan degradation, fructose and mannose metabolism, amino sugar and nucleotide sugar metabolism and galactose metabolism were annotated. These pathways were not only associated with the growth and development of petals but were also related to the synthetic substrates of anthocyanins.

Flavonoid biosynthesis, which was controlled by many genes [30], and key structural genes in the flavonoid metabolic pathway were screened in this study. Interestingly, the well-known key chalcone synthase gene (*CHS*) catalyzing the formation of the chalcone derivative could not be found, which was the committed step leading to the biosynthesis of flavonoids, isoflavonoids and anthocyanins [48]. This might be the reason that the expression of *PlCHS* in the inner-petal was not inhibited, and its level was not different with that in the outer-petal, which converted upstream materials to downstream materials in large quantity. In the expression analysis of the 10 candidate DEGs, the expression of upstream *PlCHI* was also not inhibited in the inner-petal, instead its level was higher than that in the outer-petal, which was opposite to our previous results [30], indicating that chalcone was not accumulated in the inner-petal and did not cause its yellow colour. Furthermore, the expression levels of downstream *PlDFR*, *PlANS*, *Pl3GT* and *Pl5GT* in the inner-petal were all lower compared to the outer-petal, but this difference did not reach hundreds-fold as our previous results [30], suggesting that anthocyanins were also produced in the inner-petal, just its content was relatively low.

Flower colour was mainly dependent on the kinds of pigment, the content and its distribution in the petals [49]. The generalised yellow flower from pale yellow to dark yellow could be divided into multiple grades according to its colour depth. Among these colours, the pigment of the yellow flower perceived by the naked eye was relatively complicated, which was related to flavonoids and carotenoids, this conclusion had been fully proved by previous transcriptome studies [20–23]. Previous studies on the determination of *P. lactiflora* petal pigment composition showed that a great deal of chalcone and a small amount of anthoxanthin accumulation were the cause of its yellow petal formation [27, 30]. Similarly, the biochemical mechanism of yellow characteristic formation in *P. suffruticosa* was the same, and kaempferol, quercetin, isorhamnetin, chrysoeriol, apigenin and luteolin were identified as the main anthoxanthins [50]. This was mainly because anthocyanins were responsible for red, whereas chalcones and anthoxanthins were yellow. In this study, chalcones could not be detected in the yellow inner-petal, whereas a large number of anthoxanthins (kaempferol di-hexoside, kaempferol-3-*O*-malonylglucoside-7-*O*-glucoside, quercetin-3-*O*-galactoside, luteolin-7-*O*-galloylglucoside, luteolin-7-*O*-glucoside, isorhamnetin-3-*O*-glucoside and flavone C-glycoside) and a few anthocyanins (cyanidin-3,5-di-*O*-glucoside, peonidin-3,5-di-*O*-glucoside) were identified, which was not consistent with previous results [27, 30]. Although anthocyanins were in the yellow inner-petal, the co-pigmentation effect of anthoxanthins in the inner-petal was significant and completely covered the red effect of anthocyanins due to the increase of CI from 34.00 in S1 to 69.28 in S4. In addition, compared with the red outer-petal, the contents of anthoxanthins and anthocyanins were relatively lower in the yellow inner-petal. These physiological results obtained perfect explanation using key gene expression analysis. Due to the massive expression of upstream *PlPAL* and *PlCHI*, particularly *PlCHI*, a large number of upstream materials in the inner-petal were formed and transformed to the downstream, rather than in the accumulation of chalcones. With the expression of *PlF3H*, *PlF3′H*, *PlF3′5′H* and *PlFLS*, a variety of anthoxanthins was formed and accumulated. Furthermore, a small amount of anthocyanins was produced, which was combined with low expression levels of downstream *PlDFR*, *PlANS*, *Pl3GT* and *Pl5GT*, and eventually made the inner-petal appear yellow. In addition, because the expression level of the first key gene, *PlPAL,* in the inner-petal was always lower than that in the outer-petal, combined with lower *PlFLS*, *PlDFR*, *PlANS*, *Pl3GT* and *Pl5GT* expression, the accumulation of anthoxanthins and anthocyanins in the inner-petal was lower compared to the outer-petal. Taken together, these results provided an understanding of the biochemical and molecular mechanisms of yellow characteristic formation in *P. lactiflora*, which might lay a theoretical foundation for breeding new yellow *P. lactiflora* varieties.

## Conclusion

This study showed that RNA-Seq analysis based on the high throughput sequencing technology provided an efficient approach for identifying critical genes in *P. lactiflora*. In the metabolic pathways related to yellow formation, flavonoid metabolic pathway and glucide metabolic pathway were identified, and *PlPAL*, *PlFLS*, *PlDFR*, *PlANS*, *Pl3GT* and *Pl5GT* were selected as potential candidates involved in flavonoid metabolic pathway, which induced inhibition of anthocyanin biosynthesis mediated yellow formation in *P. lactiflora*.

## Methods

### Plant materials

*P. lactiflora* was grown in the germplasm repository of Horticulture and Plant Protection College, Yangzhou University, Jiangsu Province, China (32°30′ N, 119°25′ E). A flower colour chimaera cultivar ‘Jinhui’ with red outer-petal and yellow inner-petal was used as the experimental materials, and the ground plants grew well with sufficient light and water supply. The samples were taken from March to May 2013, including four developmental stages (S1, flower-bud stage; S2, initiating bloom stage; S3, bloom stage; and S4, wither stage). Among these samples, the young outer-petal and inner-petal samples with significantly different colours were used for transcriptome sequencing. In addition, the developmental petals were used for qualitative and quantitative analysis of flavonoids and expression analysis of candidate genes. After measurement of the flower qualitative trait and colour parameter, all samples were immediately frozen in liquid nitrogen and stored at -80°C until further analysis.

### Flower qualitative trait and colour variables measurements

Flower diameter was measured by micrometer scale (Taizhou Xinshangliang Measuring Tools Co., Ltd., China). And the colour of fresh petals were compared with the RHSCC firstly [51], and then measured on a TC-P2A chroma meter (Beijing Optical Instrument Factory, China) using three colour parameters including L*, a* and b* values.

### RNA extraction, cDNA library construction and transcriptome sequencing

Total RNA was extracted according to a modified CTAB extraction protocol [25]. Prior to cDNA library construction, RNA samples were treated with DNase using DNase I kit (TaKaRa, Japan) based on the manufacturer’s instructions, and then their integrity were examined by a spectrophotometer (Eppendorf, Germany) and 1% agarose gel electrophoresis. After these, magnetic beads with Oligo (dT) were used to isolate mRNA which was fragmented into short fragments using a fragmentation buffer. Then the first cDNA strand was synthesized using the mRNA fragments as templates, and the second strand was generated using a SuperScript Double-Stranded cDNA Synthesis kit (Invitrogen, Camarillo, CA), purified via magnetic beads, the ends repaired and a single nucleotide A (adenine) added to the 3′ ends. Sequencing adaptors were then ligated to the fragments, and the suitable fragments were selected for the PCR amplification as templates. After quantification and qualification of the sample library using the Agilent 2100 Bioanalyzer (Agilent Technologies, Palo Alto, CA) and ABI StepOnePlus Real-Time PCR System (Applied Biosystems, USA), the samples were sequenced at Beijing Genomic Institute (Shenzhen, China) using an Illumina HiSeq™ 2000 platform (Illumina Inc., San Diego, CA, USA), and the workflow was as follows: template hybridization, isothermal amplification, linearization, blocking, sequencing primer hybridization, and sequencing on the sequencer for read 1. After completion of the first read, the templates could be regenerated in situ to enable a second read from the opposite end of the fragments. Once the original templates were cleaved and removed, the reverse strands underwent sequencing-by-synthesis.

### *De novo*assembly

After raw reads filtering, transcriptome *de novo* assembly was performed using the short reads assembling program Trinity, which combined three independent software modules: Inchworm, Chrysalis and Butterfly [12]. Firstly, Inchworm assembled the RNA-seq data into unique sequences of transcripts, which often generate full-length transcripts for a dominant isoform, but reported only the unique portions of alternatively spliced transcripts. Secondly, Chrysalis clustered the Inchworm Contigs into clusters and constructed complete *de* Bruijn graphs for each cluster, which represented the full transcriptional complexity for a given gene. Next, Chrysalis partitioned the full read set among these disjointed graphs. Subsequently, Butterfly processed the individual graphs in parallel, which traced the paths that reads and pairs of reads took within the graph, ultimately reporting full-length transcripts for alternatively spliced isoforms and teasing apart transcripts that corresponded to paralogous genes. The result sequences of Trinity were called Unigenes. Finally, blastx alignment (e-value < 0.00001) between Unigenes and NR (http://www.ncbi.nlm.nih.gov), Swiss-Prot (http://www.expasy.ch/sprot), KEGG (http://www.genome.jp/kegg) and COG (http://www.ncbi.nlm.nih.gov/COG) was performed, and the best aligning results were used to determine the sequence direction of Unigenes. If the results of different databases conflicted with each other, then a priority order of NR, Swiss-Prot, KEGG and COG was given to determine the sequence direction of the Unigenes. When a Unigene was unaligned with any of these databases, a software named ESTScan was used to determine its sequence direction [52]. For Unigenes with sequence directions, we provided their sequences from the 5′ end to the 3′ end; for those without any direction, we provided their sequences from the assembly software.

### Unigene annotation and analysis

Unigene annotation was performed using various bioinformatics procedures. In addition, the sequences were firstly aligned using blastx to protein databases such as NR, Swiss-Prot, KEGG and COG (e-value < 0.00001), and aligned by blastn to NT (e-value < 0.00001), thereby retrieving proteins with the highest sequence similarity with the given Unigenes along with their protein functional annotations. Secondly, the Blast2GO program [53] was used to obtain the GO annotation of Unigenes according to the NR annotation. After obtaining the GO annotation for every Unigene, WEGO software [54] was used to perform the GO functional classification for all Unigenes and to understand the distribution of the gene functions of the species on the macro-level. In addition, using the KEGG database, the complex biological behaviours of genes were further studied and pathway annotation for Unigenes was obtained by KEGG annotation.

### Unigene expression difference analysis

Unigene expression level was calculated using Fragments Per kb per Million fragments (FPKM) method [55]. DEGs were defined based on false discovery rate (FDR ≤ 0.001 and differential expression fold > 2). The confirmed DEGs were subjected to GO functional analysis and KEGG Pathway analysis.

### Gene expression analysis

Gene transcript levels were analysed using Q-PCR with a BIO-RAD CFX96™ Real-Time System (Bio-Rad, USA). The cDNA was synthesized from 1 μg RNA using PrimeScript® RT reagent Kit With gDNA Eraser (TaKaRa, Japan). *P. lactiflora Actin* (JN105299) had been used as an internal control in this study [56]. All gene-specific primers in this paper were showed in Additional file 5: Table S3. Q-PCR was performed using the SYBR® Premix Ex Taq™ (Perfect Real Time) (TaKaRa, Japan). Gene relative expression levels of target genes were calculated by the 2^-△△Ct^ comparative threshold cycle (Ct) method [57], and the expression level of *PAL* in S1 of inner-petal was used as the control. The Ct values of the triplicate reactions were gathered using the Bio-Rad CFX Manager V1.6.541.1028 software.

### Qualitative and quantitative analysis of flavonoids

Flavonoid analysis was performed according to the method of Jia et al. [27] and He et al. [58] with some modifications. The petals of each sample (1.0 g fresh weight) were extracted with 6 mL of acidic methanol solution (70: 0.1: 29.9; v/v/v, CH_3_OH: HCl: H_2_O) in a test tube and shaken in a XW-80A vortex (Shanghai Huxi Analysis Instrument Factory Co., Ltd., China) at 4°C for 24 h. The filtrate was passed through 0.22 μm membrane filters (Shanghai ANPEL Scientific Instrument Co., Ltd., China). This experiment was performed three times for each sample, and the extracts were used for analysis of the flavonoids.

Qualitative analysis of the flavonoids was performed using HPLC-ESI-MS^n^ (LCQ Deca XP MAX, Thermo). The HPLC column was TSK gel ODS-80Ts QA (4.6 mm × 250 mm) (Tosoh, Japan). The column temperature was 35°C, and the injected volume was 20 μL. The flow rate was 0.8 mL/min, and the mobile phase consisted of 10% formic acid water solution as solvent A (10:90; v/v, HCOOH:H_2_O) and methyl alcohol/acetonitrile/water (10:40:50; v/v/v, CH_3_OH:CH_3_CN:H_2_O) as solvent B. The linear gradient profile was 10% B at 0 min, 20% B at 30 min, 30% B at 50 min, 40% B at 60 min, 50% B at 65 min, 50% B at 70 min, 10% B at 75 min, and then returned to 10% B in 90 min. Analysis conditions of mass spectrometry were as follows: positive ionization mode, gas (N_2_) temperature, 300°C; nebulizer pressure, 45 Pa; sheath gas temperature, 250°C; capillary voltage, 3.5 kV; nozzle voltage, 500 V; and scan range, m/z 50–1500 units.

Quantitative analysis of the flavonoids was performed on an HPLC (Agilent 1200–6460 QQQ, USA) equipped with a Quat Pump, thermostatted Column Compartment 1260 TCC and a Diode Array Detector 1260 DAD. The chromatography analysis conditions were consistent with that of mass spectrometry. Detection was performed at 525 nm for anthocyanins, 350 nm for anthoxanthins (flavone and flavonol) and 365 nm for chalcones, and the photodiode array spectra were recorded between 200 and 800 nm. All determinations were performed with three replicates. The amount of total anthocyanins and anthoxanthins was calculated in milligrams per gram fresh weight (as a quantity of Malvidin-3,5-di-*O*-glucoside (Mv3G5G) mg g^-1^ and as a quantity of Rutin mg g^-1^, respectively).

## Electronic supplementary material

Additional file 1: Figure S1: Randomicity of *P. lactiflora* outer-petal and inner-petal reads on All-Unigene. (DOC 38 KB)

Additional file 2: Figure S2: Figures of NR annotation E-value, similarity and species distribution statistics. (DOC 112 KB)

Additional file 3: Table S1: List of pathway enriched differentially expressed genes in *P. lactiflora* outer-petal and inner-petal. (DOC 116 KB)

Additional file 4: Table S2: Identification of pigment components in *P. lactiflora* petals [59–62]. (DOC 47 KB)

Additional file 5: Table S3: Gene-specific primers sequence for detection by Q-PCR. (DOC 34 KB)

## Abbreviations

*3GT*: Anthocyanidin 3-*O*-glucosyltransferase gene
*5GT*: Anthocyanidin 5-*O*-glucosyltransferase gene
*ANS*: Anthocyanidin synthase gene
*CHI*: Chalcone isomerase gene
*CHS*: Chalcone synthase gene
CI: Co-pigment index
COG: Cluster of orthologous groups of proteins database
Ct: Threshold cycle
DEGs: Differentially expressed genes
*DFR*: Dihydroflavonol 4-reductase gene
*F3H*: Flavanone 3-hydroxylase gene
*F3′H*: Flavonoid 3′-hydroxylase gene
*F3′5′H*: Flavonoid 3′,5′- hydroxylase gene
*FLS*: Flavonol synthase gene
FPKM: Fragments per kb per million fragments
GO: Gene ontology database
HPLC-ESI-MS^n^: High-performance liquid chromatograph-electrospray ionization-mass spectrometry
KEGG: Kyoto encyclopedia of genes and genomes database
*MT*: Methyl transferase gene
NR: Non-redundant protein database
NT: Nucleotide databases
*PAL*: Phenylalanine ammonialyase gene
RHSCC: Royal horticultural society colour chart
RNA-Seq: Transcriptome sequencing
Swiss-Prot: Swiss-Prot protein database.
