# Supplementary material for: Transcriptome sequencing of a chimaera reveals coordinated expression of anthocyanin biosynthetic genes mediating yellow formation in herbaceous peony (Paeonia lactiflora Pall.)
Source: BMC Genomics. 2014 Aug 19;15(1):689. doi: 10.1186/1471-2164-15-689 (PMC4159507; doi:10.1186/1471-2164-15-689)
Supplement: Supplementary file 1 — Additional file 1: Figure S1: Randomicity of P. lactiflora outer-petal and inner-petal reads on All-Unigene. (DOC 38 KB) [file 12864_2014_6409_MOESM1_ESM.doc]

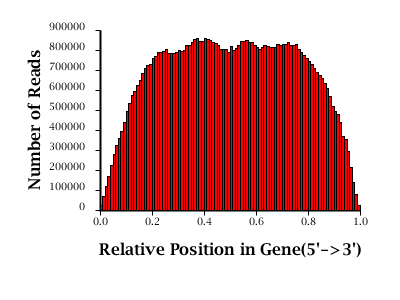

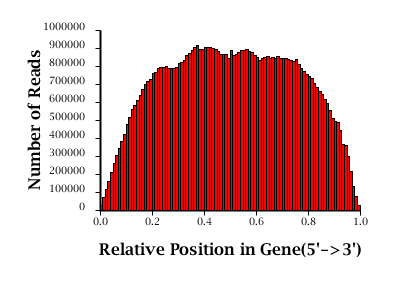


outer-petal

inner-petal

**Figure S1** **Randomicity of *P. lactiflora* outer-petal and** **inner-petal reads on All-Unigene.**
