# Supplementary figures and images for: Transcriptome sequencing of a chimaera reveals coordinated expression of anthocyanin biosynthetic genes mediating yellow formation in herbaceous peony (Paeonia lactiflora Pall.)
Source: BMC Genomics. 2014 Aug 19;15(1):689. doi: 10.1186/1471-2164-15-689 (PMC4159507; doi:10.1186/1471-2164-15-689)

**
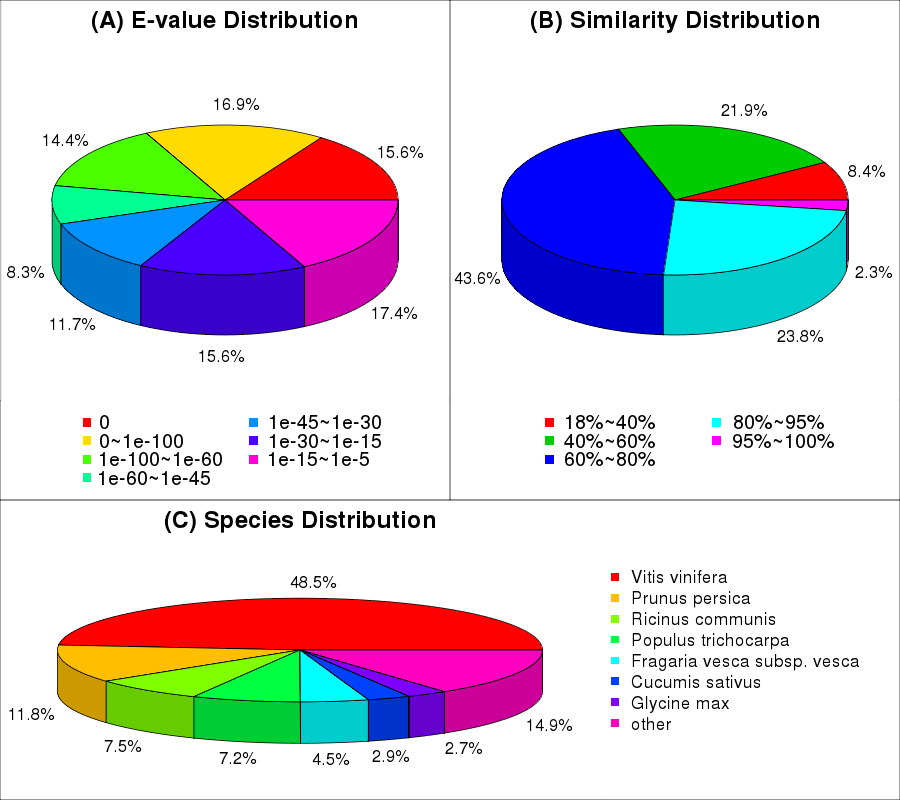
**

**Figure S2 Figures of NR annotation E-value, similarity and species distribution statistics.**

Supplement: Supplementary file 2 — Additional file 2: Figure S2: Figures of NR annotation E-value, similarity and species distribution statistics. (DOC 112 KB) [file 12864_2014_6409_MOESM2_ESM.doc]
