# Supplementary material for: Transcriptome sequencing of a chimaera reveals coordinated expression of anthocyanin biosynthetic genes mediating yellow formation in herbaceous peony (Paeonia lactiflora Pall.)
Source: BMC Genomics. 2014 Aug 19;15(1):689. doi: 10.1186/1471-2164-15-689 (PMC4159507; doi:10.1186/1471-2164-15-689)
Supplement: Supplementary file 3 — Additional file 3: Table S1: List of pathway enriched differentially expressed genes in P. lactiflora outer-petal and inner-petal. (DOC 116 KB) [file 12864_2014_6409_MOESM3_ESM.doc]

**Table S1 List of pathway enriched differentially expressed genes in *P. lactiflora* outer-petal and inner-petal**

| No. | Pathway | DEGs with pathway annotation (2,559) | All genes with pathway annotation (20,294) | Pathway ID |
| --- | --- | --- | --- | --- |
| 1 | [Metabolic pathways](../../../../C:%5CDocuments%20and%20Settings%5CZHAO%5C%E6%A1%8C%E9%9D%A2%5CF13TSFECKF0882_PAEwxiT%5Cdiff_exp%5CPathway%5COuter-petal-vs-Inner-petal.htm#gene1%23gene1) | 796 (31.11%) | 4523 (22.29%) | ko01100 |
| 2 | [Pentose and glucuronate interconversions](../../../../C:%5CDocuments%20and%20Settings%5CZHAO%5C%E6%A1%8C%E9%9D%A2%5CF13TSFECKF0882_PAEwxiT%5Cdiff_exp%5CPathway%5COuter-petal-vs-Inner-petal.htm#gene2%23gene2) | 92 (3.6%) | 217 (1.07%) | ko00040 |
| 3 | [Starch and sucrose metabolism](../../../../C:%5CDocuments%20and%20Settings%5CZHAO%5C%E6%A1%8C%E9%9D%A2%5CF13TSFECKF0882_PAEwxiT%5Cdiff_exp%5CPathway%5COuter-petal-vs-Inner-petal.htm#gene3%23gene3) | 145 (5.67%) | 491 (2.42%) | ko00500 |
| 4 | [Ether lipid metabolism](../../../../C:%5CDocuments%20and%20Settings%5CZHAO%5C%E6%A1%8C%E9%9D%A2%5CF13TSFECKF0882_PAEwxiT%5Cdiff_exp%5CPathway%5COuter-petal-vs-Inner-petal.htm#gene4%23gene4) | 121 (4.73%) | 379 (1.87%) | ko00565 |
| 5 | [Biosynthesis of secondary metabolites](../../../../C:%5CDocuments%20and%20Settings%5CZHAO%5C%E6%A1%8C%E9%9D%A2%5CF13TSFECKF0882_PAEwxiT%5Cdiff_exp%5CPathway%5COuter-petal-vs-Inner-petal.htm#gene5%23gene5) | 429 (16.76%) | 2197 (10.83%) | ko01110 |
| 6 | [Glycerophospholipid metabolism](../../../../C:%5CDocuments%20and%20Settings%5CZHAO%5C%E6%A1%8C%E9%9D%A2%5CF13TSFECKF0882_PAEwxiT%5Cdiff_exp%5CPathway%5COuter-petal-vs-Inner-petal.htm#gene6%23gene6) | 142 (5.55%) | 518 (2.55%) | ko00564 |
| 7 | [Endocytosis](../../../../C:%5CDocuments%20and%20Settings%5CZHAO%5C%E6%A1%8C%E9%9D%A2%5CF13TSFECKF0882_PAEwxiT%5Cdiff_exp%5CPathway%5COuter-petal-vs-Inner-petal.htm#gene7%23gene7) | 146 (5.71%) | 583 (2.87%) | ko04144 |
| 8 | [Carotenoid biosynthesis](../../../../C:%5CDocuments%20and%20Settings%5CZHAO%5C%E6%A1%8C%E9%9D%A2%5CF13TSFECKF0882_PAEwxiT%5Cdiff_exp%5CPathway%5COuter-petal-vs-Inner-petal.htm#gene8%23gene8) | 53 (2.07%) | 140 (0.69%) | ko00906 |
| 9 | [Flavone and flavonol biosynthesis](../../../../C:%5CDocuments%20and%20Settings%5CZHAO%5C%E6%A1%8C%E9%9D%A2%5CF13TSFECKF0882_PAEwxiT%5Cdiff_exp%5CPathway%5COuter-petal-vs-Inner-petal.htm#gene9%23gene9) | 39 (1.52%) | 94 (0.46%) | ko00944 |
| 10 | [Photosynthesis - antenna proteins](../../../../C:%5CDocuments%20and%20Settings%5CZHAO%5C%E6%A1%8C%E9%9D%A2%5CF13TSFECKF0882_PAEwxiT%5Cdiff_exp%5CPathway%5COuter-petal-vs-Inner-petal.htm#gene10%23gene10) | 17 (0.66%) | 23 (0.11%) | ko00196 |
| 11 | [Phenylpropanoid biosynthesis](../../../../C:%5CDocuments%20and%20Settings%5CZHAO%5C%E6%A1%8C%E9%9D%A2%5CF13TSFECKF0882_PAEwxiT%5Cdiff_exp%5CPathway%5COuter-petal-vs-Inner-petal.htm#gene11%23gene11) | 82 (3.2%) | 322 (1.59%) | ko00940 |
| 12 | [Cutin, suberine and wax biosynthesis](../../../../C:%5CDocuments%20and%20Settings%5CZHAO%5C%E6%A1%8C%E9%9D%A2%5CF13TSFECKF0882_PAEwxiT%5Cdiff_exp%5CPathway%5COuter-petal-vs-Inner-petal.htm#gene12%23gene12) | 29 (1.13%) | 75 (0.37%) | ko00073 |
| 13 | [Limonene and pinene degradation](../../../../C:%5CDocuments%20and%20Settings%5CZHAO%5C%E6%A1%8C%E9%9D%A2%5CF13TSFECKF0882_PAEwxiT%5Cdiff_exp%5CPathway%5COuter-petal-vs-Inner-petal.htm#gene13%23gene13) | 36 (1.41%) | 108 (0.53%) | ko00903 |
| 14 | [Oxidative phosphorylation](../../../../C:%5CDocuments%20and%20Settings%5CZHAO%5C%E6%A1%8C%E9%9D%A2%5CF13TSFECKF0882_PAEwxiT%5Cdiff_exp%5CPathway%5COuter-petal-vs-Inner-petal.htm#gene14%23gene14) | 76 (2.97%) | 319 (1.57%) | ko00190 |
| 15 | [Stilbenoid, diarylheptanoid and gingerol biosynthesis](../../../../C:%5CDocuments%20and%20Settings%5CZHAO%5C%E6%A1%8C%E9%9D%A2%5CF13TSFECKF0882_PAEwxiT%5Cdiff_exp%5CPathway%5COuter-petal-vs-Inner-petal.htm#gene15%23gene15) | 41 (1.6%) | 137 (0.68%) | ko00945 |
| 16 | [Plant-pathogen interaction](../../../../C:%5CDocuments%20and%20Settings%5CZHAO%5C%E6%A1%8C%E9%9D%A2%5CF13TSFECKF0882_PAEwxiT%5Cdiff_exp%5CPathway%5COuter-petal-vs-Inner-petal.htm#gene16%23gene16) | 189 (7.39%) | 1062 (5.23%) | ko04626 |
| 17 | [Flavonoid biosynthesis](../../../../C:%5CDocuments%20and%20Settings%5CZHAO%5C%E6%A1%8C%E9%9D%A2%5CF13TSFECKF0882_PAEwxiT%5Cdiff_exp%5CPathway%5COuter-petal-vs-Inner-petal.htm#gene17%23gene17) | 43 (1.68%) | 157 (0.77%) | ko00941 |
| 18 | [Glycolysis / Gluconeogenesis](../../../../C:%5CDocuments%20and%20Settings%5CZHAO%5C%E6%A1%8C%E9%9D%A2%5CF13TSFECKF0882_PAEwxiT%5Cdiff_exp%5CPathway%5COuter-petal-vs-Inner-petal.htm#gene18%23gene18) | 62 (2.42%) | 276 (1.36%) | ko00010 |
| 19 | [Photosynthesis](../../../../C:%5CDocuments%20and%20Settings%5CZHAO%5C%E6%A1%8C%E9%9D%A2%5CF13TSFECKF0882_PAEwxiT%5Cdiff_exp%5CPathway%5COuter-petal-vs-Inner-petal.htm#gene19%23gene19) | 26 (1.02%) | 87 (0.43%) | ko00195 |
| 20 | [Benzoxazinoid biosynthesis](../../../../C:%5CDocuments%20and%20Settings%5CZHAO%5C%E6%A1%8C%E9%9D%A2%5CF13TSFECKF0882_PAEwxiT%5Cdiff_exp%5CPathway%5COuter-petal-vs-Inner-petal.htm#gene20%23gene20) | 16 (0.63%) | 41 (0.2%) | ko00402 |
| 21 | [Anthocyanin biosynthesis](../../../../C:%5CDocuments%20and%20Settings%5CZHAO%5C%E6%A1%8C%E9%9D%A2%5CF13TSFECKF0882_PAEwxiT%5Cdiff_exp%5CPathway%5COuter-petal-vs-Inner-petal.htm#gene21%23gene21) | 8 (0.31%) | 12 (0.06%) | ko00942 |
| 22 | [Zeatin biosynthesis](../../../../C:%5CDocuments%20and%20Settings%5CZHAO%5C%E6%A1%8C%E9%9D%A2%5CF13TSFECKF0882_PAEwxiT%5Cdiff_exp%5CPathway%5COuter-petal-vs-Inner-petal.htm#gene22%23gene22) | 44 (1.72%) | 186 (0.92%) | ko00908 |
| 23 | [Other glycan degradation](../../../../C:%5CDocuments%20and%20Settings%5CZHAO%5C%E6%A1%8C%E9%9D%A2%5CF13TSFECKF0882_PAEwxiT%5Cdiff_exp%5CPathway%5COuter-petal-vs-Inner-petal.htm#gene23%23gene23) | 30 (1.17%) | 110 (0.54%) | ko00511 |
| 24 | [Plant hormone signal transduction](../../../../C:%5CDocuments%20and%20Settings%5CZHAO%5C%E6%A1%8C%E9%9D%A2%5CF13TSFECKF0882_PAEwxiT%5Cdiff_exp%5CPathway%5COuter-petal-vs-Inner-petal.htm#gene24%23gene24) | 156 (6.1%) | 927 (4.57%) | ko04075 |
| 25 | [Isoflavonoid biosynthesis](../../../../C:%5CDocuments%20and%20Settings%5CZHAO%5C%E6%A1%8C%E9%9D%A2%5CF13TSFECKF0882_PAEwxiT%5Cdiff_exp%5CPathway%5COuter-petal-vs-Inner-petal.htm#gene25%23gene25) | 16 (0.63%) | 46 (0.23%) | ko00943 |
| 26 | [ABC transporters](../../../../C:%5CDocuments%20and%20Settings%5CZHAO%5C%E6%A1%8C%E9%9D%A2%5CF13TSFECKF0882_PAEwxiT%5Cdiff_exp%5CPathway%5COuter-petal-vs-Inner-petal.htm#gene26%23gene26) | 49 (1.91%) | 227 (1.12%) | ko02010 |
| 27 | [Ascorbate and aldarate metabolism](../../../../C:%5CDocuments%20and%20Settings%5CZHAO%5C%E6%A1%8C%E9%9D%A2%5CF13TSFECKF0882_PAEwxiT%5Cdiff_exp%5CPathway%5COuter-petal-vs-Inner-petal.htm#gene27%23gene27) | 31 (1.21%) | 126 (0.62%) | ko00053 |
| 28 | [Fructose and mannose metabolism](../../../../C:%5CDocuments%20and%20Settings%5CZHAO%5C%E6%A1%8C%E9%9D%A2%5CF13TSFECKF0882_PAEwxiT%5Cdiff_exp%5CPathway%5COuter-petal-vs-Inner-petal.htm#gene28%23gene28) | 31 (1.21%) | 126 (0.62%) | ko00051 |
| 29 | [Nitrogen metabolism](../../../../C:%5CDocuments%20and%20Settings%5CZHAO%5C%E6%A1%8C%E9%9D%A2%5CF13TSFECKF0882_PAEwxiT%5Cdiff_exp%5CPathway%5COuter-petal-vs-Inner-petal.htm#gene29%23gene29) | 24 (0.94%) | 93 (0.46%) | ko00910 |
| 30 | [Amino sugar and nucleotide sugar metabolism](../../../../C:%5CDocuments%20and%20Settings%5CZHAO%5C%E6%A1%8C%E9%9D%A2%5CF13TSFECKF0882_PAEwxiT%5Cdiff_exp%5CPathway%5COuter-petal-vs-Inner-petal.htm#gene30%23gene30) | 46 (1.8%) | 222 (1.09%) | ko00520 |
| 31 | [alpha-Linolenic acid metabolism](../../../../C:%5CDocuments%20and%20Settings%5CZHAO%5C%E6%A1%8C%E9%9D%A2%5CF13TSFECKF0882_PAEwxiT%5Cdiff_exp%5CPathway%5COuter-petal-vs-Inner-petal.htm#gene31%23gene31) | 26 (1.02%) | 109 (0.54%) | ko00592 |
| 32 | [Cyanoamino acid metabolism](../../../../C:%5CDocuments%20and%20Settings%5CZHAO%5C%E6%A1%8C%E9%9D%A2%5CF13TSFECKF0882_PAEwxiT%5Cdiff_exp%5CPathway%5COuter-petal-vs-Inner-petal.htm#gene32%23gene32) | 31 (1.21%) | 141 (0.69%) | ko00460 |
| 33 | [Galactose metabolism](../../../../C:%5CDocuments%20and%20Settings%5CZHAO%5C%E6%A1%8C%E9%9D%A2%5CF13TSFECKF0882_PAEwxiT%5Cdiff_exp%5CPathway%5COuter-petal-vs-Inner-petal.htm#gene33%23gene33) | 30 (1.17%) | 142 (0.7%) | ko00052 |
| 34 | [Diterpenoid biosynthesis](../../../../C:%5CDocuments%20and%20Settings%5CZHAO%5C%E6%A1%8C%E9%9D%A2%5CF13TSFECKF0882_PAEwxiT%5Cdiff_exp%5CPathway%5COuter-petal-vs-Inner-petal.htm#gene34%23gene34) | 16 (0.63%) | 63 (0.31%) | ko00904 |
| 35 | [Sesquiterpenoid and triterpenoid biosynthesis](../../../../C:%5CDocuments%20and%20Settings%5CZHAO%5C%E6%A1%8C%E9%9D%A2%5CF13TSFECKF0882_PAEwxiT%5Cdiff_exp%5CPathway%5COuter-petal-vs-Inner-petal.htm#gene35%23gene35) | 13 (0.51%) | 48 (0.24%) | ko00909 |
| 36 | [Glutathione metabolism](../../../../C:%5CDocuments%20and%20Settings%5CZHAO%5C%E6%A1%8C%E9%9D%A2%5CF13TSFECKF0882_PAEwxiT%5Cdiff_exp%5CPathway%5COuter-petal-vs-Inner-petal.htm#gene36%23gene36) | 27 (1.06%) | 130 (0.64%) | ko00480 |
| 37 | [Ribosome](../../../../C:%5CDocuments%20and%20Settings%5CZHAO%5C%E6%A1%8C%E9%9D%A2%5CF13TSFECKF0882_PAEwxiT%5Cdiff_exp%5CPathway%5COuter-petal-vs-Inner-petal.htm#gene37%23gene37) | 117 (4.57%) | 745 (3.67%) | ko03010 |
