# Supplementary material for: Transcriptome sequencing of a chimaera reveals coordinated expression of anthocyanin biosynthetic genes mediating yellow formation in herbaceous peony (Paeonia lactiflora Pall.)
Source: BMC Genomics. 2014 Aug 19;15(1):689. doi: 10.1186/1471-2164-15-689 (PMC4159507; doi:10.1186/1471-2164-15-689)
Supplement: Supplementary file 4 — Additional file 4: Table S2: Identification of pigment components in P. lactiflora petals [59–62]. (DOC 47 KB) [file 12864_2014_6409_MOESM4_ESM.doc]

**Table S2** **Identification of pigment components in *P. lactiflora* petals**

| Peak | Retention time  (min) | λ_max_ (nm) | [M+H]^+^ (m/z) | MS^2^ (m/z) | Tentative identification | Reference |
| --- | --- | --- | --- | --- | --- | --- |
| a1 | 6.12 | 260, 510 | 611.20 | 448.90, 287.20 | Cyanidin-3,5-di-*O*-glucoside | [34] |
| a2 | 9.48 | 275, 515 | 625.20 | 462.90, 301.10 | Peonidin-3,5-di-*O*-glucoside | [34] |
| f1 | 9.52 | 265, 345 | 611.10 | 449.10, 287.30 | Kaempferol di-hexoside | [34] |
| f2 | 11.91 | 265, 345 | 697.00 | 448.89, 287.27 | Kaempferol-3- *O*-malonylglucoside-7-*O*-glucoside | [29] |
| f3 | 21.44 | 265, 360 | 465.13 | 302.0 | Quercetin-3-*O*-galactoside | [35] |
| f4 | 24.68 | 275, 365 | 601.00 | 287.21 | Luteolin-7-*O*-galloylglucoside | [29] |
| f5 | 28.23 | 265, 365 | 449.00 | 287.00 | Luteolin-7-*O*-glucoside | [36] |
| f6 | 30.90 | 265, 350 | 479.00 | 317.00 | Isorhamnetin-3-*O*-glucoside | [35] |
| f7 | 32.42 | 265, 360 | 535.00 | 463.00, 445.00 | Flavone C-glycoside | [37] |
