# Supplementary material for: Transcriptome sequencing of a chimaera reveals coordinated expression of anthocyanin biosynthetic genes mediating yellow formation in herbaceous peony (Paeonia lactiflora Pall.)
Source: BMC Genomics. 2014 Aug 19;15(1):689. doi: 10.1186/1471-2164-15-689 (PMC4159507; doi:10.1186/1471-2164-15-689)
Supplement: Supplementary file 5 — Additional file 5: Table S3: Gene-specific primers sequence for detection by Q-PCR. (DOC 34 KB) [file 12864_2014_6409_MOESM5_ESM.doc]

| Gene | Forward primer (5'-3') | Reverse primer (5'-3') |
| --- | --- | --- |
| *Actin* | GCAGTGTTCCCCAGTATT | TCTTTTCCATGTCATCCC |
| *PAL* | ACATTCTCGCCACTACCA | CTTCCGAAATTCCTCCAC |
| *CHI* | TCCCACCTGGTTCTTCTA | AACTCTGCTTTGCTTCCG |
| *F3H* | AGTTCTTCGCTTTACCGC | CAATCTCGCACAGCCTCT |
| *F3'H* | TGGCTACTACATTCCAAAAG | CCAAACGGTATAACCTCAA |
| *F3'5'H* | AGTCTTGTGGGTGGGTTT | TGTTTCTGTCGGTCATCTTA |
| *FLS* | GGCTTCTTCCTCGTCACT | CCCTTCAAACTTTCCTCTAC |
| *DFR* | CTTCCTGTGGAAAAGAACC | CCAAAAACAAACCAGAGATC |
| *ANS* | AGGAGAAGATCATACTCAAG | ACAAAGAAGCACAAAGGCAC |
| *F3GT* | AACACCGAATGCCTAAAC | AGCCACCCATCACTAAAT |
| *F5GT* | GAAGCGTCTCTGTTTTACC | CTCCTTGTCTCCATCTCG |

**Table S3 Gene-speciﬁc primers sequence for detection by Q-PCR**
